# The Effects of Atorvastatin on Global Cerebral Ischemia-Induced Neuronal Death

**DOI:** 10.3390/ijms22094385

**Published:** 2021-04-22

**Authors:** A Ra Kho, Dae Ki Hong, Beom Seok Kang, Woo-Jung Park, Kyung Chan Choi, Kyoung-Ha Park, Sang Won Suh

**Affiliations:** 1 Department of Physiology, College of Medicine, Hallym University, Chuncheon 24252, Korea; arakho136@naver.com (A.R.K.); zxnm01220@gmail.com (D.K.H.); ttiger1993@gmail.com (B.S.K.); 2 Division of Cardiovascular Disease, Hallym University Medical Center, Anyang 14068, Korea; cathpark@hallym.or.kr; 3 Department of Pathology, Chuncheon Sacred Heart Hospital, College of Medicine, Hallym University, Chuncheon 24252, Korea; kcchoi@hallym.ac.kr

**Keywords:** global cerebral ischemia, neuronal death, oxidative stress, inflammation, vasa vasorum, endothelial cell damage

## Abstract

(1) Background and Purpose: Global cerebral ischemia-induced severe hypoxic brain damage is one of the main causes of mortality and long-term neurologic disability even after receiving early blood reperfusion. This study aimed to test the hypothesis that atorvastatin potentially has neuroprotective effects in global cerebral ischemia (GCI). (2) Methods: We performed two sets of experiments, analyzing acute (1-week) and chronic (4-week) treatments. For the vehicle (Veh) and statin treatments, 1 mL of 0.9% saline and 5 mg/kg of atorvastatin (ATOR) were administered orally. For histological analysis, we used the following staining protocols: Fluoro-Jade B and NeuN, 4-hydroxynonenal, CD11b and GFAP, IgG, SMI71, and vWF. Finally, we evaluated the cognitive function with a battery of behavioral tests. (3) Results: The GCI-ATOR group showed significantly reduced neuronal death, oxidative stress, inflammation, and BBB disruption compared with the GCI-Veh group. Moreover, the GCI-ATOR group showed decreased endothelial damage and VV proliferation and had significantly improved cognitive function compared with the GCI-Veh group in both models. (4) Conclusions: ATOR has neuroprotective effects and helps recover the cognitive function after GCI in rats. Therefore, administration of atorvastatin may be a therapeutic option in managing GCI after CA.

## 1. Introduction

Global cerebral ischemia (GCI) commonly occurs in patients who have a cardiac arrest (CA) [1,2]. The primary determinant of the clinical outcome of CA is hypoxic brain injury. Therefore, GCI is not only a major primary cause of death but is also associated with significant neurologic disability after CA [3,4]. Clinically, GCI is distinct from focal cerebral ischemia (FCI), which is often associated with atherosclerotic or embolic stroke [5]. Initial neuroprotective management is especially important in GCI, and therapeutic induction of hypothermia is known to improve neurologic recovery after severe cerebral ischemia [6]. Despite rigorous research and management, the outcomes of GCI associated with CA have not appreciably changed [7,8].

Statin is a well-known lipid-lowering agent and has long been known to exert vascular protective and pleiotropic effects, such as anti-inflammatory, anti-oxidant, and immunologic modulation, thereby reducing neuronal damage following FCI [9,10,11,12]. In addition, statins induce angiogenesis, neurogenesis, and synaptogenesis and inhibit neuro-inflammation after FCI [13,14,15]. The current guidelines recommend the prescription of statins for the secondary prevention of FCI [16].

Vasa vasorum (VV) are a constitute a network of microvessels that plays a nutritive role in the adventitial walls of arteries. VV proliferates in pathologic conditions such as atherosclerosis or ischemic change [17]. The mechanism by which hypoxia contributes to the development of VV may involve the activation of inflammatory processes, which in turn stimulate the angiogenic growth factors that promote the neovascularization of the VV [18]. This phenomenon suggests that the VV serve as a conduit for the entry of pro-inflammatory and pro-atherosclerotic components into the artery wall [19,20].

Studies have reported that statin has beneficial effects in FCI, which is mainly associated with atherosclerotic cardiovascular disease. However, the roles and benefits of statin in GCI have not yet been reported. In addition, no research on the association between statin and VV neovascularization after GCI has been reported. Therefore, this study aimed to investigate the neuroprotective effects of statin and the neovascularization of VV in a CGI rat model.

## 2. Results

The groups of subjects evaluated in this study were as follows: Sham-Veh, Sham-ATOR, GCI-Veh, and GCI-ATOR; one set was evaluated for the acute (1-week) treatment, and another set for the chronic (4-week) treatment, for a total of eight study groups.

### 2.1. Atorvastatin Reduces Neuronal Death after Global Cerebral Ischemia

**Figure 1:**
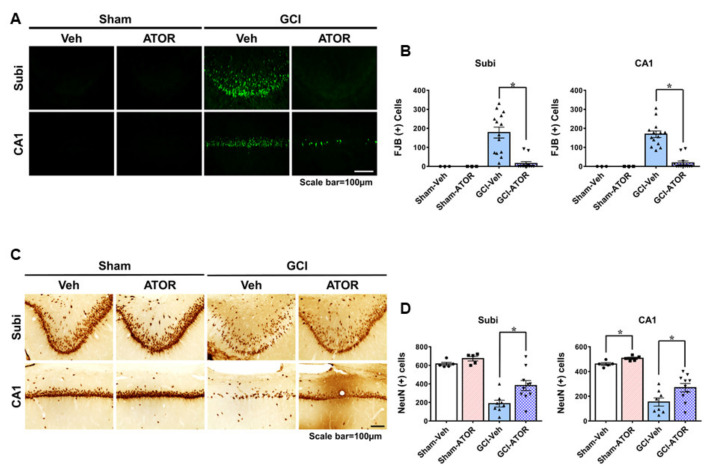
Atorvastatin reduces neuronal death after global cerebral ischemia. Global ischemia induced neuronal death in the subiculum and CA1 of the hippocampus region 1 and 4 weeks after ischemia. Fluoro-Jade B-positive neurons in Subi and CA1 (**A**). Oral post-treatment with atorvastatin (5 mg/kg) for 1 week demonstrated neuroprotective effects after ischemia. Live neurons in Subi and CA1 (**C**). Oral post-treatment with atorvastatin (5 mg/kg) for 4 weeks demonstrated neuroprotective effects after ischemia. Atorvastatin greatly decreased hippocampal neuronal death compared with the vehicle treatment. Scatter dot plot bar graphs represent the quantified degenerating and live neurons (**B**,**D**). Data are the mean ± S.E.M.; for the acute model, n = 3 for each sham group. n = 12–14 for each ischemia group. For the chronic model, n = 5 for each sham group, n = 8–10 for each ischemia group. * Significantly different from the vehicle-treated group, * *p* < 0.05.

To investigate whether ATOR exerts neuroprotective effects on GCI, we performed FJB staining to access neuronal degeneration after 1 week and we performed NeuN staining to detect live neurons after 4 weeks in the hippocampal subiculum (Subi) and in the cornu ammonis 1 (CA1) region. In the sham-operated groups, no FJB-positive cells were found, while the number of degenerating neurons was increased in Subi and CA1 after GCI (Figure 1A). Figure 1B shows that neuronal death was significantly reduced in the GCI-ATOR group compared with the GCI-Veh group. NeuN staining, a robust marker that can identify live neurons, revealed the presence of numerous live neurons in the sham-operated groups and the loss of a considerable number of live neurons in the GCI-Veh group [21,22,23,24,25]. However, the GCI-ATOR group had more live neurons in Subi and CA1 than the GCI-Veh group (Figure 1C,D) (*p* < 0.05).

### 2.2. Atorvastatin Reduces Ischemia-Induced Oxidative Stress

**Figure 2:**
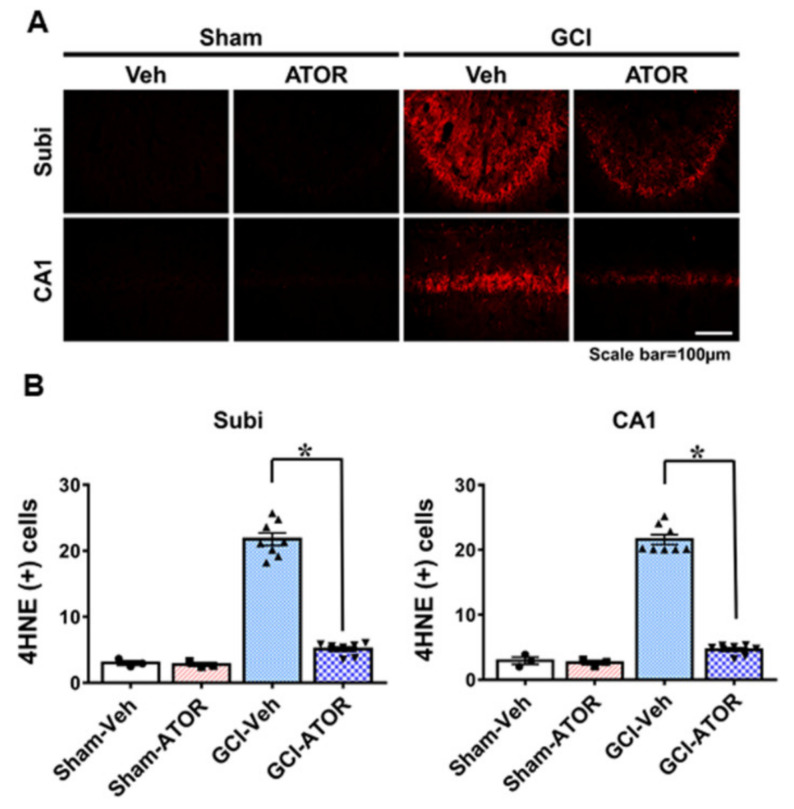
Atorvastatin reduces ischemia-induced oxidative stress. Neuronal oxidative stress was detected through 4HNE (red color) staining in CA1 and subiculum 1 week after ischemia. The sham-operated groups showed a weak red fluorescence in hippocampal CA1 and subiculum. The GCI-ATOR group showed a weaker 4HNE fluorescence intensity compared with the GCI-Veh group (**A**). Scatter dot plot bar graph represents the quantified 4HNE intensity in each region (**B**). Data are mean ± S.E.M.; n = 3 for each sham group, n = 8 for each ischemia group. * Significantly different from the vehicle-treated group, * *p* < 0.05.

To estimate the degree of oxidative damage after GCI, we performed 4HNE staining. The brains were stained with a 4HNE antibody 1 week after GCI to determine whether oxidative stress was caused by GCI and whether ATOR administration reduced oxidative stress causing hippocampal neuronal death. No 4HNE fluorescence intensity was observed in either Sham-Veh and Sham-ATOR groups. By contrast, the intensity of 4HNE staining in the Subi and CA1 regions of the hippocampus was significantly increased in the GCI-Veh group, whereas it was greatly reduced in the GCI-ATOR group (Figure 2). As shown in Figure 2B, the GCI-ATOR group showed a slightly reduced 4HNE staining intensity in the Subi and CA1 regions compared with the GCI-Veh group (Figure 2B).

### 2.3. Atorvastatin Reduces Microglia and Astrocyte Activation after Global Cerebral Ischemia

**Figure 3:**
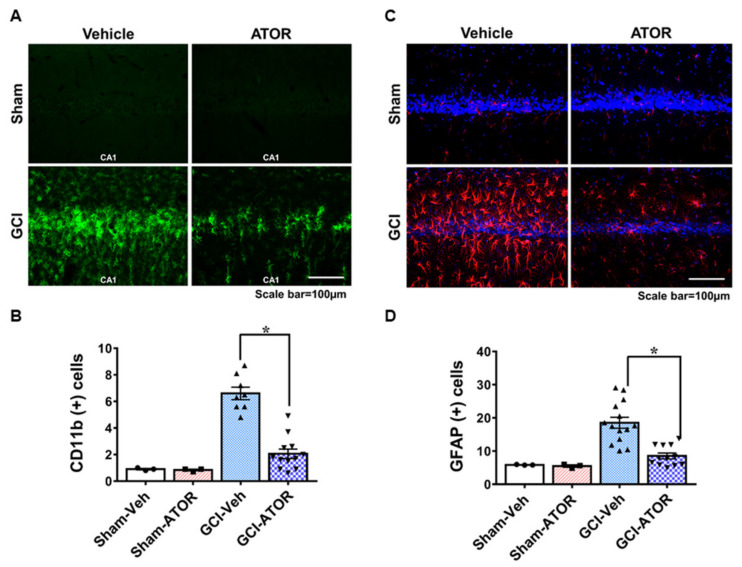
Atorvastatin reduces microglia and astrocyte activation after global cerebral ischemia. GCI induces an excessive inflammatory response by promoting microglia and astrocyte activation in the damaged area. The 1-week atorvastatin treatment prevented inflammation in CA1 following GCI. Activation of microglia (**A**) and astrocytes (**C**) in the sham-operated and GCI-induced rats. Scatter dot plot bar graphs represent the degree of microglia activation and the intensity of activated astrocytes in the CA1 region (**B**,**D**). Data are mean ± S.E.M., n = 3 for each sham group, n = 8 for each ischemia group. * Significantly different from the vehicle-treated group, * *p* < 0.05.

To confirm the activation of glial cells, we stained the brain samples harvested at 1 week after GCI with CD11b and GFAP and determined the degree of microglia and astrocyte activation in the four groups. The sham-operated groups had resting microglia and small numbers of astrocytes. Relative to the number of activated microglia and astrocytes in the CA1 region in the sham-operated groups, that in the GCI-Veh group was significantly increased. However, microglia and astrocyte activation in the GCI-ATOR group was significantly decreased compared with that in the GCI-Veh group (Figure 3).

### 2.4. Atorvastatin Prevents Blood–Brain Barrier Disruption after Global Cerebral Ischemia

**Figure 4:**
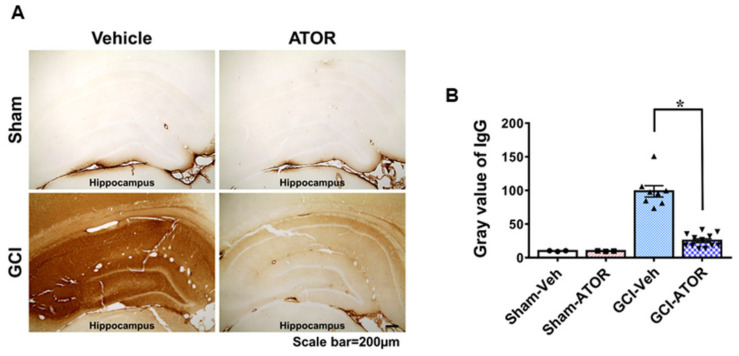
Atorvastatin prevents blood–brain barrier disruption after global cerebral ischemia. Due to ischemic damage, BBB was disrupted, as seen from the high concentration of serum IgG that leaked out through damaged vessels. In the sham-operated group, no IgG leakage was observed because the BBB remained intact; in the ischemia-induced group, the BBB was destroyed, and IgG consequently leaked from the damaged vessels, which was confirmed by a chemical reaction (**A**). The degree of IgG extravasation decreased in the GCI-ATOR group, indicating the protective effects of atorvastatin towards the BBB. Scatter dot plot bar graph representing the degree of IgG leakage in the whole hippocampus (**B**). Data are mean ± S.E.M., n = 3 for each sham group, n = 8~12 for each ischemia group. * Significantly different from the vehicle-treated group, * *p* < 0.05.

To assess the breakdown of the BBB, the extent of serum IgG leakage from the damaged vessel was evaluated via IgG staining. A slight IgG leakage was observed in the sham-operated group. Meanwhile, the GCI-Veh group showed a significantly increased IgG leakage compared with the GCI-ATOR group. Figure 4 shows that a meaningful difference in the concentration gradient of IgG leakage was found between the sham-operated and the GCI groups. In the vehicle-treated groups, we confirmed that IgG leaked due to the destruction of vessels after GCI, and the brain tissues were visibly darkened in response to IgG antibodies. Nevertheless, ATOR administration greatly reduced ischemia-induced IgG leakage (Figure 4).

### 2.5. Atorvastatin Prevents Endothelial Cell Loss and Vasa Vasorum Proliferation after Global Cerebral Ischemia

**Figure 5:**
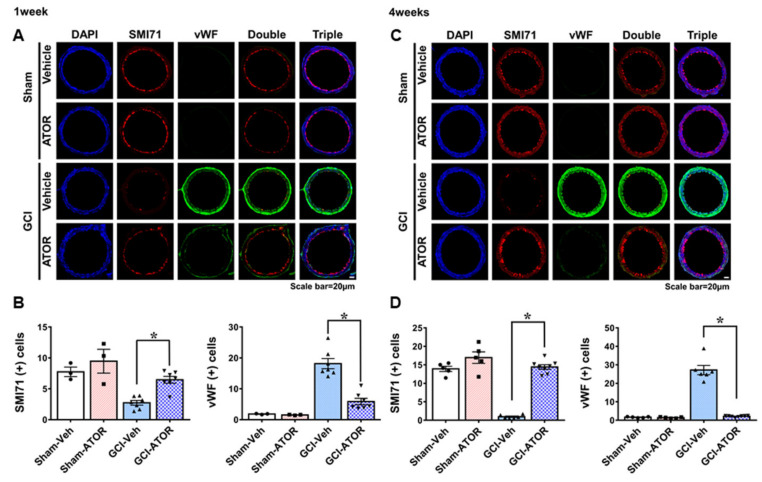
Atorvastatin prevents endothelial cell loss and vasa vasorum proliferation after global ischemia. Treatment with atorvastatin for 1-week after GCI decreased endothelial cell loss and restrained vasa vasorum proliferation (**A**). The 4-week atorvastatin treatment after GCI considerably reduced endothelial cell loss and significantly inhibited vasa vasorum proliferation (**C**). Scatter dot plot bar graphs showing the quantification of endothelial cells and vWF levels at 1 and 4 weeks (**B**,**D**, respectively). Data are mean ± S.E.M.; for both acute and chronic model, n = 3–5 for each sham group, n = 6–8 for each ischemia group. * Significantly different from the vehicle-treated group, * *p* < 0.05.

To estimate the loss of endothelial cells and the change in VV neovascularization in the internal carotid artery after GCI, we performed SMI71 and vWF staining (Figure 5). Firstly, SMI71 staining showed that the endothelial cells in the sham-Veh and sham-ATOR groups were normally positioned. In the GCI-Veh group, endothelial cell loss was caused by ischemic injury. However, in the GCI-ATOR group, endothelial cell loss after ischemia was greatly reduced. In addition, vWF staining visualized VV proliferation. VV proliferation was rarely observed in the sham-Veh and sham-ATOR groups in both the 1- and 4-week treatment groups, whereas it was significantly increased in the GCI-Veh group. By contrast, in the GCI-ATOR group, VV proliferation was significantly reduced (Figure 5).

### 2.6. Atorvastatin Improves the Cognitive Function after Global Cerebral Ischemia

**Figure 6:**
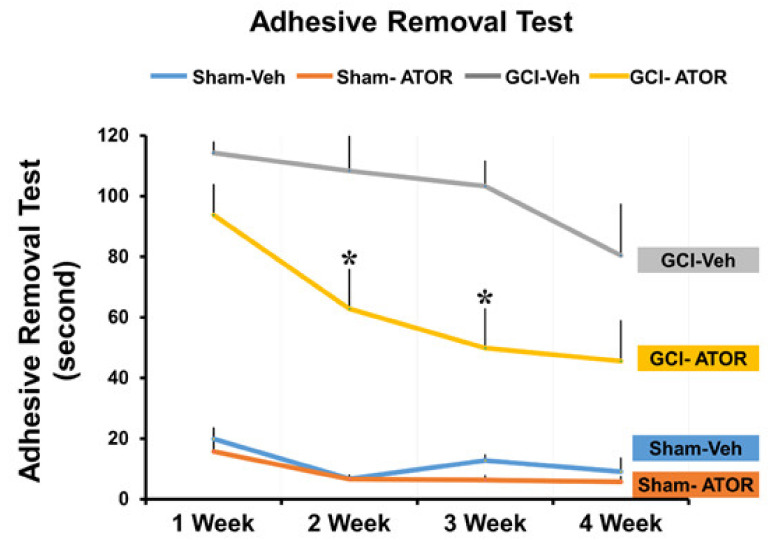
Atorvastatin improves the cognitive function after global cerebral ischemia. The groups treated with atorvastatin for 4 weeks were evaluated for changes in cognitive and sensory function through a series of adhesive removal tests, and the results are graphically presented. Compared with the GCI-Veh group, the GCI-ATOR group showed significantly improved cognitive impairment. Data are mean ± S.E.M., n = 5 for each sham group, n = 8–10 for each ischemia group. * Significantly different from the vehicle-treated group, * *p* < 0.05.

To evaluate cognitive impairment, we conducted standard adhesive removal tests every week on the same day for 4 weeks after surgery. As shown in Figure 6, the rats in the GCI-induced group failed to remove the tape within 120 s or were not successful for nearly 90 s. However, as time passed, the rats in the GCI-ATOR group in the chronic model were able to remove the tapes more quickly, that is, in less than 1 min.

## 3. Discussion

In this study, we showed that ATOR reduces neuronal death, ischemia-induced oxidative stress, inflammatory response, and BBB breakdown, as well as improves the cognitive function after GCI. In addition, ATOR prevents endothelial cell loss and inhibits VV proliferation in the rat internal carotid artery after GCI. This study demonstrated for the first time that ATOR has neuroprotective effects and that it reduces VV neovascularization in a GCI rat model.

GCI occurs when blood flow to the brain is stopped or reduced, which is usually triggered by CA or refractory cardiogenic shock. Despite the advances in therapeutic approaches over the past several decades, GCI continues to be the leading cause of morbidity and mortality after CA [2,8]. Many investigators have attempted to reduce neurologic injury after GCI either by increasing cerebral blood flow or by reducing the cerebral metabolic rate of oxygen consumption. While reperfusion may be essential in protecting the brain, it may also lead to reperfusion injury that results from the restoration of blood supply to an ischemic brain tissue. Therefore, the enthusiasm for elucidating the post-ischemic cerebral circulatory dynamics has declined. To the present, neurotoxic theories on post-ischemic brain injury appear more promising than theories based on post-ischemic hypo-perfusion [1].

Studies have demonstrated that statins effectively prevent primary and secondary strokes. Experimental studies on FCI have shown that, apart from lipid-lowering effects, statins suppress neurological cell death through anti-inflammatory and antioxidant effects, inhibition of the immune response, antithrombotic effects, and endothelial nitric oxide synthetase upregulation [26,27,28,29,30]. Moreover, surrogate marker studies have suggested that the benefits of statins are mediated by the generation of collateral vessels and by enhanced reperfusion, as well as by reduced infarct size, as shown in an experimental acute FCI study [31,32]. Another study demonstrated that the protective effect of statins on acute cerebral ischemia was lost two to four days after the withdrawal of statin treatment [33]. In addition, research has suggested that statin administration immediately after stroke (≤72 h) improves clinical outcomes [34]. The international guidelines recommend that patients suffering from acute FCI should be treated with statins for secondary prevention [16,35]. However, there is no guideline for using statins in global cerebral ischemia.

In this study, we confirmed that ATOR significantly reduced ischemia-induced neuronal death. The results of the FJB staining indicated that the number of degenerating neurons in the Subi and CA1 regions in the GCI-ATOR group decreased compared to those in the GCI-Veh group. This finding is consistent with the NeuN staining results, demonstrating that ATOR has a neuroprotective effect on GCI. Studies have shown that a damage caused by neurological disorders, such as ischemia, hypoglycemia, and traumatic brain injury, causes p47^phox^ to over-activate NADPH oxidases and induces the production of reactive oxygen species, resulting in oxidative stress damage in the hippocampus [36,37,38]. Our study confirmed that oxidative damage occurred after GCI and that oxidative damage significantly decreased following treatment with ATOR, which demonstrated its antioxidant effects.

The concept regarding the physiological function of neuroglial cells for substance exchange and for providing metabolic support to neurons remains quite valid to this day. Studies have shown that by becoming activated via insult, disruption of the elaborate balance among neurons, astrocytes, and microglia contributes to more severe neurodegeneration or other neurological diseases [39]. Under hypoxic/ischemic conditions, astrocytes and microglia can be excessively activated by neurotoxic substances or pro-inflammatory factors, leading to neuroinflammation [40]. Brain inflammation has been considered a potential target to treat stroke, and various approaches have been employed to suppress ischemia-induced brain inflammation. In particular, the CA1 region of the hippocampus appeared vulnerable to the activation of reactive astrocyte and microglia in our GCI animal model. Many papers have already reported that ATOR demonstrates acute anti-inflammatory effects in cardiovascular diseases [41,42]. In the present study, we confirmed that ATOR administration reduces microglia and astrocyte activation, resulting in a reduction in neuronal death after GCI.

The BBB maintains a biological and anatomical mechanism that controls the exchange of substances between the brain and the blood. It plays an important role in maintaining the homeostasis of the brain microenvironment and in helping neurons to function properly [43]. Under ischemic conditions, activated glial and endothelial cells produce cytokines and chemokines that induce lymphocyte migration across the BBB as well as enhance the permeability of the brain blood vessels; as a result, brain edema severely worsens, leading to neurological disorders [44,45]. Rivaze et al. mentioned that ATOR protects the BBB by upregulating the activity of tight junction proteins, such as ZO-1 and occludin, in the endothelial tight junctions, consequently limiting the permeability of the BBB [46]. In the present study, we performed IgG staining to detect IgG leakage and found that the degree of BBB disruption was considerably lower in the group administered ATOR than in the group not treated with it after GCI.

VV is constitute a network of microvessels that play a nutritive role in the walls of arteries. While the inner portions of a vessel wall are nourished by the diffusion of luminal blood, the outer portions of a wall often fall outside of this diffusional range and thus require supplementary nourishment from the VV [17,47]. In pathologic conditions such as atherosclerosis or ischemic change, proliferation of VV occurs. Recent studies have demonstrated that VV play an important role in the transport of vascular inflammatory markers, such as Rho-kinase, and ultimately in the decrease in microvascular function [48,49]. Studies involving rat carotid artery injury models have reported that the number of VV, particularly in the adventitia, was increased and was accompanied by VEGF expression [50,51]. In our study, to estimate the loss of endothelial cells and the change in VV neovascularization in the internal carotid artery, we performed SMI71 and vWF staining. First, endothelial cell loss was significantly reduced in the group treated with ATOR after ischemia. In addition, an increased adventitial vWF staining was observed in the GCI-Veh group, an indication of VV proliferation. By contrast, the GCI-ATOR group showed decreased adventitial VV neovascularization. Therefore, considering that VV becomes a conduit of pro-inflammatory and pre-atherosclerotic components, proliferation can be presumed to be associated with increased neuronal and vascular damage after GCI. Our observations suggest that controlling the neovascularization of the VV may be a potential therapeutic target in GCI.

Several potential limitations of our study should be considered. First, the GCI was not directly induced by CA in the rats. However, systemic shock was induced by a blood pressure of less than 40–50 mmHg; moreover, a 7 min bilateral carotid artery clamping was performed, and isoelectric EEG was confirmed; thus, GCI was believed to have been induced in a manner sufficiently like in situ CA. Second, the long-term effects of statin on neuroprotection and VV neovascularization in GCI were not investigated because of the limited lifespan of the rats. Third, only high-dose ATOR was evaluated in this study, and changes in ATOR doses were not evaluated. Fourth, we did not obtain a clear pathologic finding on VV neovascularization in the rat internal carotid artery using hematoxylin and eosin staining. Therefore, more extensive animal experiments with a focus on a range of statin doses, along with human clinical trials, are needed to confirm the present observations.

## 4. Materials and Methods

### 4.1. Animals

Eight-week-old adult male Sprague-Dawley rats (290–330 g) were purchased from DBL Corporation (Chungcheongbuk-do, Eumseong-gun, Korea). To evaluate the neuroprotective effects of statin after GCI, we performed two sets of experiments involving: acute (1-week) and chronic (4-week) treatments. The experimental protocols for this study were approved by the National Institutes of Health and were conducted in accordance with the guidelines of the Committee on Animal Use for Study and Education of Hallym University (Protocol No. Hallym 2018-7 (26.04.2018)). All rats were given free access to food and water, were kept in a temperature-controlled (20 ± 2 °C) and humidity-controlled (55 ± 5%) vivarium. Room lights were controlled automatically, turned on and off in a 12 h light/dark cycle.

### 4.2. Global Cerebral Ischemia Surgery

Transient GCI animal models were induced using the method described by Smith et al. [52]. The rats were anesthetized with 2–3% isoflurane in 70% NO_2_/balanced O_2_ via nose cone. The body temperature was maintained at 37 °C ± 1.0 °C using a heating pad and a heating lamp. A catheter was inserted into the femoral artery to check the blood pressure and to obtain blood samples. We clamped the common carotid artery that we found and then extracted blood using a heparin syringe, keeping the average systemic arterial pressure at 40–50 mmHg, while maintaining the body temperature at 37 °C; the carotid artery was placed back in when the ischemia induction was completed. Successful induction of GCI was verified through electroencephalograph (EEG) (BIOPAC System Inc., Santa Brabara, CA, USA) monitoring [36,53]. Bilateral burr holes were made in the temporal areas of the skull, and then EEG probes were inserted beneath the dura. A reference needle was placed in the neck muscle, and the isoelectric EEG signal was observed for 7 min. Rats in the sham ischemia group underwent the same surgical procedure but without blood extraction and the clamping of the common carotid arteries.

### 4.3. Statin Administration

To evaluate the effects of statin on neuronal death after GCI, we performed atorvastatin (ATOR, Pfizer pharmaceuticals, Vega Baja, PR, USA) treatments in two sets of experiments involving acute (1-week) and chronic (4-week) treatments. For the acute model, the global cerebral ischemia-vehicle (GCI-Veh, n = 14) and the sham operation-vehicle groups (Sham-Veh, n = 5) were given 1 mL of 0.9% saline for 1 week. By contrast, the sham operated-atorvastatin group (Sham-ATOR, n = 5) and the global cerebral ischemia-atorvastatin group (GCI-ATOR, n = 12) received ATOR (5 mg/kg, p.o., 1 week). In the chronic model, the rats in the GCI-Veh (n = 8) and Sham-Veh (n = 5) were given 1 mL of 0.9% saline for 4 weeks, whereas the Sham-ATOR (n = 5) and the GCI-ATOR (n = 10) rats received ATOR (5 mg/kg, p.o., 4 weeks). All rats in the acute and chronic groups were sacrificed at 1 and 4 weeks after GCI, respectively.

### 4.4. Brain Sample Procedure

The animals were sacrificed 1 and 4 weeks after GCI, using urethane (1.5 g/kg, i.p.) in order to deeply anesthetize animals. The brains were perfused transcardially with 0.9% saline and then fixed with 4% paraformaldehyde in phosphate-buffered saline. The brains were post-fixed in the same fixative for 1 h and then immersed in 30% sucrose for cryoprotection until the brains sank. When the brains sank completely to the bottom of the sucrose solution, the entire brains were frozen and subsequently sectioned coronally into 30 μm-thick slices with a cryostat; the brain sections were kept in a storage solution until histological evaluation.

### 4.5. Analysis of Hippocampal Neuronal Death

To investigate the degenerating neurons after GCI, brain sections (30 μm-thick) were put on gelatin-coated slides for Fluoro-Jade B (FJB) staining according to the method described by Hopkins and Schmued [54]. The slides were sequentially immersed in the following solutions: (1) 100% alcohol for 3 min; (2) 70% alcohol for 1 min; (3) distilled water for washing; (4) 0.06% potassium permanganate for 15 min; (5) 0.001% FJB (Histo-Chem Inc., Jefferson, AR, USA) solution for 30 min; (6) distilled water for washing, three times for 10 min each round. Subsequently, the brain samples were dried, covered with a cover slide, and observed under a fluorescence microscope using blue (450–490 nm) excitation light. To quantify the degree of neuronal death, we chose five coronal brain sections obtained starting at 4.0 mm posterior to Bregma and collected each section at 75 μm intervals until 5 sections were collected. A blinded experimenter counted the total number of FJB cells in the same area (magnification = 10×) of the hippocampal subiculum (900 × 1200 µm) and CA1 (900 × 400 µm). The average total number of degenerating neurons in each region was used for statistical assessment.

### 4.6. Immunohistochemistry

To block the endogenous peroxidase activity, we immersed the brain sections in 1.2% hydrogen peroxide for 20 min at room temperature. After washing in PBS, the brain sections were incubated with a mouse monoclonal anti-NeuN antibody (diluted 1:500; Millipore, Cambridge, UK) in phosphate-buffered saline with 0.3% Triton X-100 overnight at 4 °C to quantify the live neurons after GCI. Subsequently, after washing in PBS, the brain sections were incubated in biotinylated anti-mouse IgG secondary antibody (diluted 1:250; Vector Laboratories, Burlingame, CA, USA) for 2 h at room temperature and then washed again. Then, the sections were immersed in ABC solution (Burlingame, Vector, CA, USA) for 2 h at room temperature on a shaker and then washed. The immune reaction was visualized with 3,3′-diaminobenzidine solution (0.06% DAB agar, Sigma-Aldrich Co., St. Louis, MO, USA) in 0.01 M PBS buffer (100 mL) and 30% hydrogen peroxide (50 µL) for 1 min, and immunoreactions were checked. Finally, the brain samples were placed on gelatin-coated slides, and the live neurons were observed under an Axioscope microscope (Carl Zeiss, München-Hallbergmoos, Germany) [55].

To confirm blood–brain barrier (BBB) disruption, we performed IgG staining per the routine immunostaining protocols as above. The brain sections were incubated with biotinylated anti-mouse IgG secondary antibody (diluted 1:250; Vector Laboratories, Burlingame, CA, USA) for 2 h at room temperature. After washing, the sections were immersed in ABC solution for 2 h and then immunized with 3,3′-diaminobenzidine solution for 3 min. The following subsequent steps were similar to those previously described. For NeuN, we quantified the number of live neurons on the same way as for FJB analysis. For IgG, to quantify the IgG leakage area, the image was loaded into Image J (select the menu option Image → Type → 8 bit and then, edit → invert). To measure the area, the menu option Analyze → Measure was selected, and the area was recorded. We used mean gray value in our analysis.

### 4.7. Immunofluorescence Analysis

Immunofluorescence staining was performed per the routine immunostaining protocols. The primary antibodies used in this study were as follows: rabbit anti-4HNE (diluted 1:500; Alpha Diagnostic Intl. Inc., San Antonio, Texas, USA), mouse anti-CD11b (diluted 1:500; Bio-Rad, Berkeley, CA, USA), goat anti-GFAP (diluted 1:100; Abcam, Cambridge, UK), mouse anti-SMI71 (diluted 1:500; Covance, Princeton, New Jersey, USA), and von Willebrand factor (vWF; diluted 1:200; Abcam). After the incubation with the primary antibodies, the secondary antibodies were added. For 4HNE, CD11b, GFAP, SMI71, and vWF, fluorescent-conjugated secondary antibodies were used (diluted 1:250; Invitrogen. Waltham, MA, USA). The brain sections were counterstained with 4,6-diamidino-2-phenylindole (DAPI; diluted 1:1000; Invitrogen). The fluorescence-stained samples were placed on gelatin-coated slides, mounted using a mixture of distyrene, a plasticizer, and xylene (DPX mounting medium, Sigma-Aldrich, Munich, Germany), and then covered with cover slides. For 4HNE, GFAP, SMI71 and vWF, to quantify the intensity, the images were loaded into Image J v. 1.47c. Then, the following menu options were selected: Image → Adjust → Color to threshold and darken background. Subsequently, brightness was adjusted in accordance with the intensity of oxidative stress. The image was converted to an 8-bit image file by selecting the menu option Image → Type → 8 bit. To measure the area, the menu option Analyze → Measure was selected. We used mean gray value in our analysis. For CD11b staining, we used widely accepted functional standards of microglial activation: (A) morphology score of 0: no activated morphology (amoeboid morphology with enlarged soma and thickened processes); 1: 1–45% of microglia; 2: 45–90% of microglia; and 3: >90% of microglia with activated morphology; (B) we used the following 2 scores to analyze CD11b-immunoreactivity: cell number score of 0: no cells are present; 1: 1–9 cells; 2: 10–20 cells; and 3: >20 cells with continuous processes per 100 µm^2^; (C) intensity score of 0: no expression; 1: weak expression; 2: average expression; and 3: intense expression. The total score is the sum the three scores (microglial morphology, cell number, and intensity), ranging from zero to nine (magnification = 20×) [56,57].

### 4.8. Behavioral Testing

Starting on the day after the GCI, adhesive removal tests were performed once a week for 4 weeks to verify whether the oral administration of ATOR improved the cognitive function after injury. After being given time to adapt to the conditions in a transparent test box (45 × 35 × 20 cm), the rats were gently removed in order to attach 1 × 1 cm stickers onto the palm of each of their forepaws. Then, we immediately placed the rats into the test box and determined the time it took them to remove the stickers form their paws. Five trials were performed in each session, and each rat was given 1 min break between trials. A maximum of 120 s was allocated for each trial. The maximum time was recorded whenever a rat failed to remove the sticker within 120 s.

### 4.9. Data Analysis

Data are presented as mean values ± S.E.M., between-group comparisons were performed using analysis of variance (ANOVA) in accordance with the Bonferroni post-hoc test. Differences were regarded as statistically significant at *p* < 0.05.

## 5. Conclusions

ATOR has neuroprotective effects and helps in maintaining the cognitive function after GCI. Moreover, ATOR apparently inhibits VV proliferation, suggesting statins as a therapeutic option in managing GCI after CA.

## Data Availability

The datasets generated during and/or analyzed during the current study are available from the corresponding authors on reasonable request.
